# Carrion from large carnivores and food from humans subsidize mesocarnivores year round

**DOI:** 10.1038/s41598-025-15503-w

**Published:** 2025-08-14

**Authors:** Mauriel Rodriguez Curras, Mark C. Romanski, Jonathan N. Pauli

**Affiliations:** 1 Department of Forest & Wildlife Ecology, University of Wisconsin-Madison, Madison, WI USA; 2 Isle Royale National Park, US National Park Service, Houghton, MI USA; 3 Present Address: Department of Environmental Science, Policy, and Management, University of California-Berkeley, Berkeley, CA USA

**Keywords:** Carnivora, Human-wildlife conflict, Interspecific conflict, Intraguild predation, Meso-predator release, Non-consumptive impacts, Ecology, Restoration ecology, Stable isotope analysis

## Abstract

The return of large carnivores is predicted to suppress meso-carnivores, though it has only been observed in a minority of contemporary studies. Isle Royale is a remote island wilderness in Lake Superior, USA, managed for outdoor recreation. Following their natural extirpation, the National Parks Service translocated gray wolves (*Canis lupus*) in 2018–2019 and we expected the return of suppression and trophic facilitation of meso-carnivores. Nevertheless, we hypothesized that human resource subsidies led to a breakdown in meso-predator release. From Fall 2021-Winter 2024, we captured 16 individual red foxes (*Vulpes vulpes*), collected whiskers, and subsampled them to recreate the yearly diet of the population using stable isotope analysis. Notably, meso-carnivores were not demographically suppressed. Coinciding with the dates when Isle Royale National Park opened, fox diets were generalized, dissimilar between individuals, and primarily composed of human foods (0.28 ± 0.02) during summer. In winter, fox diets were specialized and exhibited high similarity, being composed primarily of carrion subsidized by wolves (0.62 ± 0.04). We propose that the complementarity of human resource subsidies to communities and ecosystems, broadly, may help explain the limited reach of the anticipated interactions following the return of large carnivores, such as meso-predator release.

**Supplementary Information:**

The online version contains supplementary material available at 10.1038/s41598-025-15503-w.

## Introduction

Over the past millennia, the world’s largest predators have been extirpated from up to 99% of their historical ranges^1^. In their absence, researchers have found support for the meso-predator release hypothesis – the emergence and predominance of mid-sized predators that have become ecologically released from competitive suppression by superordinate predators^2^. Since its development, the meso-predator release hypothesis has been demonstrated within terrestrial carnivores (Order: Carnivora^3,4^; and linked to body-size mediated competitive interactions, whereby larger carnivores (especially those 2-4x larger) competitively suppress subordinate species^3,5^. Today, large carnivores are returning to much of their historical ranges^6,7^ and, under meso-predator release, researchers and managers anticipate that the restoration of large carnivores will bring with it the horizontal interactions that limit meso-carnivores^2,8^. Importantly, though, large carnivores are returning to novel landscapes modified by humans^9^, featuring a greater distribution of the wildlife-urban interface^10^, altered species assemblages^11^, and a panoply of prey and human food subsidies^12^. As a result, we have not observed a widespread suppression of meso-carnivores even with restored apex carnivores^13^; indeed, a recent review found that only about one quarter of studies have found support for the meso-predator release hypothesis following the return of apex carnivores in Europe^14^. Even in remote and relatively undisturbed environments, the reintroduction of large carnivores does not always result in meso-carnivore suppression^15^. The question remains, then, why have we not seen meso-predator release broadly following the return of large carnivores?

Along with suppression of meso-carnivores, large carnivores also provide important resource subsidies, not only to obligate^16^, but to facultative scavengers, including the same meso-carnivores they suppress^17,18^. While subsidies from large carnivores are mediated by bottom-up resource pulses during the growing season^19^ and phenological punctuations^20,21^, such subsidies can support meso-carnivore populations during times of resource scarcity^22^. Indeed, the seasonal resource pulse that define northern ecosystems – resource abundance in summer and scarcity in winter – creates a predictable syncopation of resources for scavenging meso-carnivores^22^.

**Fig. 1:**
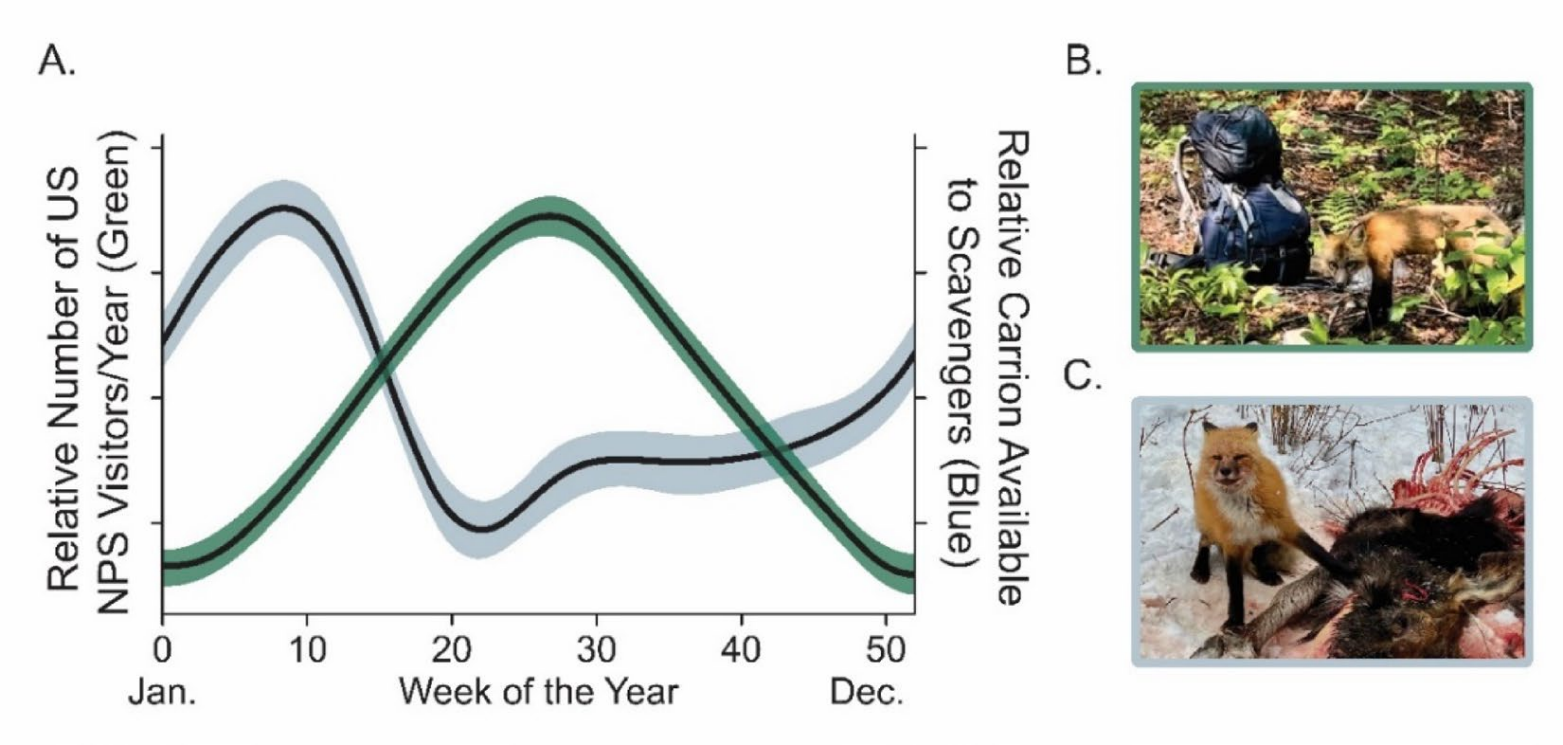
Illustration of the contrasting seasonal pattern of potential human resource subsidies (the number of United States National Park visitors per year; green) and carrion availability to scavengers (e.g., the red fox *Vulpes vulpes;* blue; (**A**). Data on carrion available to scavengers was obtained from Wilmers and Getz (2004 Ecological Modeling) following the wolf reintroduction to Yellowstone National Park. Example of a red fox visiting a hiker’s pack during summer (**B**) and a red fox scavenging from a wolf-killed moose in winter (C; Photo credits: B, Mauriel Rodriguez Curras and C, Brenna Cassidy).

Increasingly, human food subsidies – in the form of agriculture, domestic livestock and pets, bird seed, hunting bait, and human refuse – are altering trophic facilitation in ecosystems globally^23–26^. These subsidies have wide-ranging effects and have been shown to alter competition within carnivore communities^12^ and the co-existence dynamics between competing carnivores^27,28^. Much like trophic facilitation by large carnivores, human food subsidies are spatially and temporally dynamic^29^. For example, human-derived food resources in cities are ubiquitous throughout the year and coupled with the lack of large carnivores, lead to high levels of meso-carnivore abundances and diverse co-occurring carnivores^30,31^. Human food subsidies also exhibit temporal changes in both rural and natural systems. Rural agricultural landscapes feature crop harvesting and livestock calving, while public lands and natural areas feature human visitation and recreation which generally occur during summer (Fig. 1). Indeed, meso-carnivores have been shown to cue in on these resource pulses, altering their diet and interspecific interactions^29,32^.

Meso-carnivores are especially responsive to resource subsidies^17^. In winter, meso-carnivores often rely on carrion subsidized by large carnivores (Pereira et al. 2014), though this resource can create a “fatal attraction”^33^, whereby the resource pulse is accompanied by heightened risk of encounter with larger carnivores^34,35^. In summer, human subsidies create an alternative (additive) resource pulse for meso-carnivores, which has been shown to pose a low immediate risk and can lead to increased survival and reproduction^27^. Nevertheless, studies that incorporate both carrion facilitation from large carnivores and human resource subsidies in structuring the diets of meso-carnivores remain limited, particularly in relatively natural landscapes limited to recreation. Our previous work in Isle Royale showed a lack of demographic consequences to meso-carnivores following a large carnivore reintroduction, which we attributed to changes in the consumption of human foods^15^. Accordingly, we postulated that humans alter trophic facilitation to meso-predators, which may tip the scales of suppression and facilitation and lead to the break-down of meso-predator release.

Isle Royale is an isolated, wilderness archipelago in the western reaches of Lake Superior, USA and home to the longest predator-prey (wolf *Canis lupus*-moose *Alces alces*) study on record^36^. Wolves went functionally extinct in the last decade on Isle Royale^15,37^, which culminated in the reintroduction of nineteen wolves from 2018 to 2019^38^. Following the reintroduction of wolves to Isle Royale, the restoration of vertical interactions (i.e., predation on moose and beaver) was observed^39^, but surprisingly the horizontal effects to meso-carnivores (red foxes; *Vulpes vulpes*) were transient and fox populations rebounded to their pre-wolf baseline quickly after the wolves returned^15^. Foxes have been previously observed scavenging moose kills from wolves and consuming human foods during the summer (Fig. 1), but the relative importance of these food items and the seasonality of their consumption remains unknown.

Herein, we tested a potential mechanism by which meso-predator release may break down following the functional return of large carnivores by quantifying the seasonal diet of red foxes (*Vulpes vulpes*) in Isle Royale National Park using a combination of scat and stable isotope analysis to estimate the diets of foxes throughout the year. We hypothesized that the seasonality of meso-carnivore foraging is mediated by resource subsidies. Specifically, we predicted that wolves provided carrion subsidies to red foxes, especially during times of low resource availability in winter. Alternatively, we predicted that humans provide resource subsidies to foxes during summer, which would lead to individuals developing alternative foraging strategies. Our work in a relatively undisturbed wilderness limited to recreation highlights an important mechanism by which meso-predator release may break down in contemporary landscapes – contributing to the accelerated rise of the meso-predator following the return of large carnivores, even in protected landscapes.

## Results

**Fig. 2:**
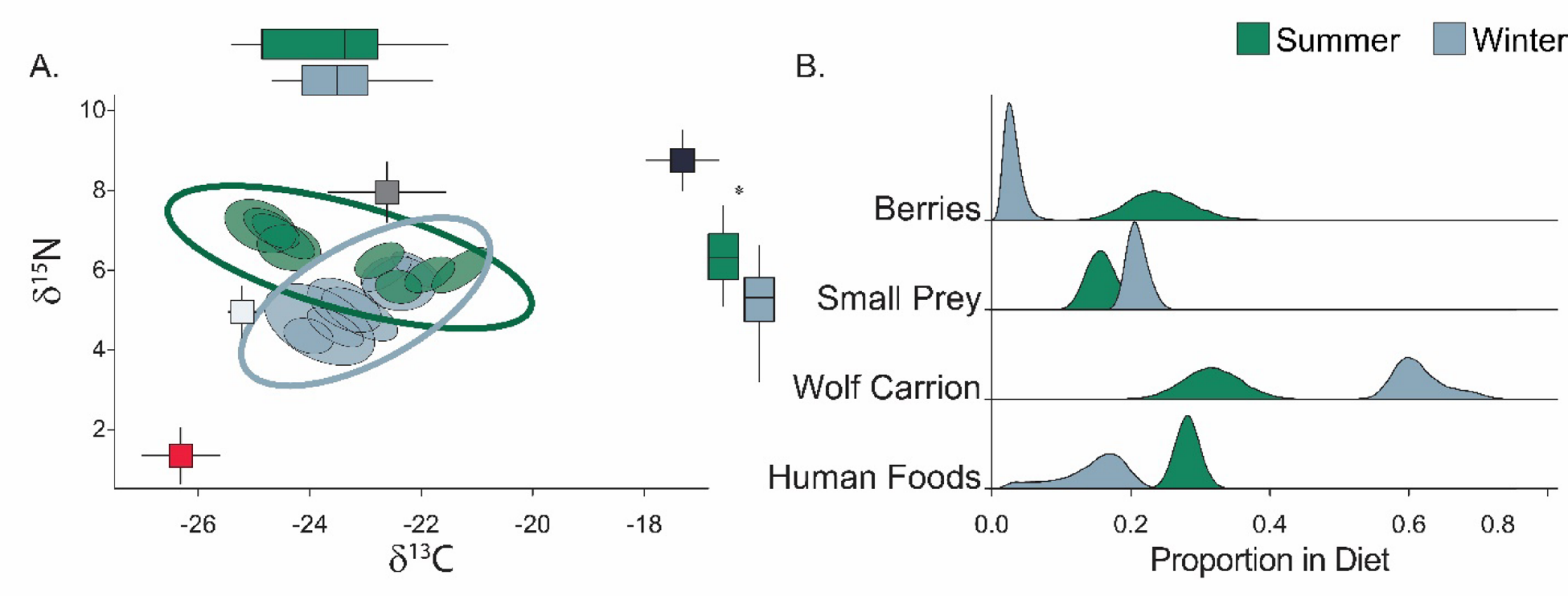
(**A**) Standard ellipse areas of red fox (*Vulpes vulpes*) isotopic niche quantified by δ^13^C and δ^15^N at the population- (ellipse outlines) and individual- (filled ellipses) level during summer (green) and winter (blue) featuring four putative prey groups available to individuals (berries – red, small prey – dark grey, wolf carrion – light grey, and human foods – black). The associated box plots along the x- and y-axes show the differences between δ^13^C and δ^15^N across seasons. (**B**) Proportion of assimilated diets (of our prey groups Berries, Small Prey, Wolf Carrion, and Human Foods) of red foxes during summer (green) and winter (blue) from our informed mixing model.

We analyzed the longitudinal diet of 16 individual red foxes on Isle Royale (N_Summer_=8 and N_Winter_=8) using stable isotope analysis along whisker segments to quantify the year-round diet of the population (SI Table 2). As expected, the trophic niche breadth (SEA) of foxes was broader in summer (2.71 ± 0.40‰^2^) compared to winter (1.88 ± 0.30‰^2^; *p* < 0.01; Fig. 2A), and the trophic niche overlap between foxes in summer and winter was low (0.30 ± 0.04). This shift in the trophic niche space of foxes appeared to be primarily driven by the nitrogen isotopic composition (δ^15^N) of foxes: individual foxes were enriched in nitrogen in summer (6.33 ± 0.70‰) compared to winter (5.21 ± 0.83‰; *p* < 0.01) Fig. 2A).

**Fig. 3:**
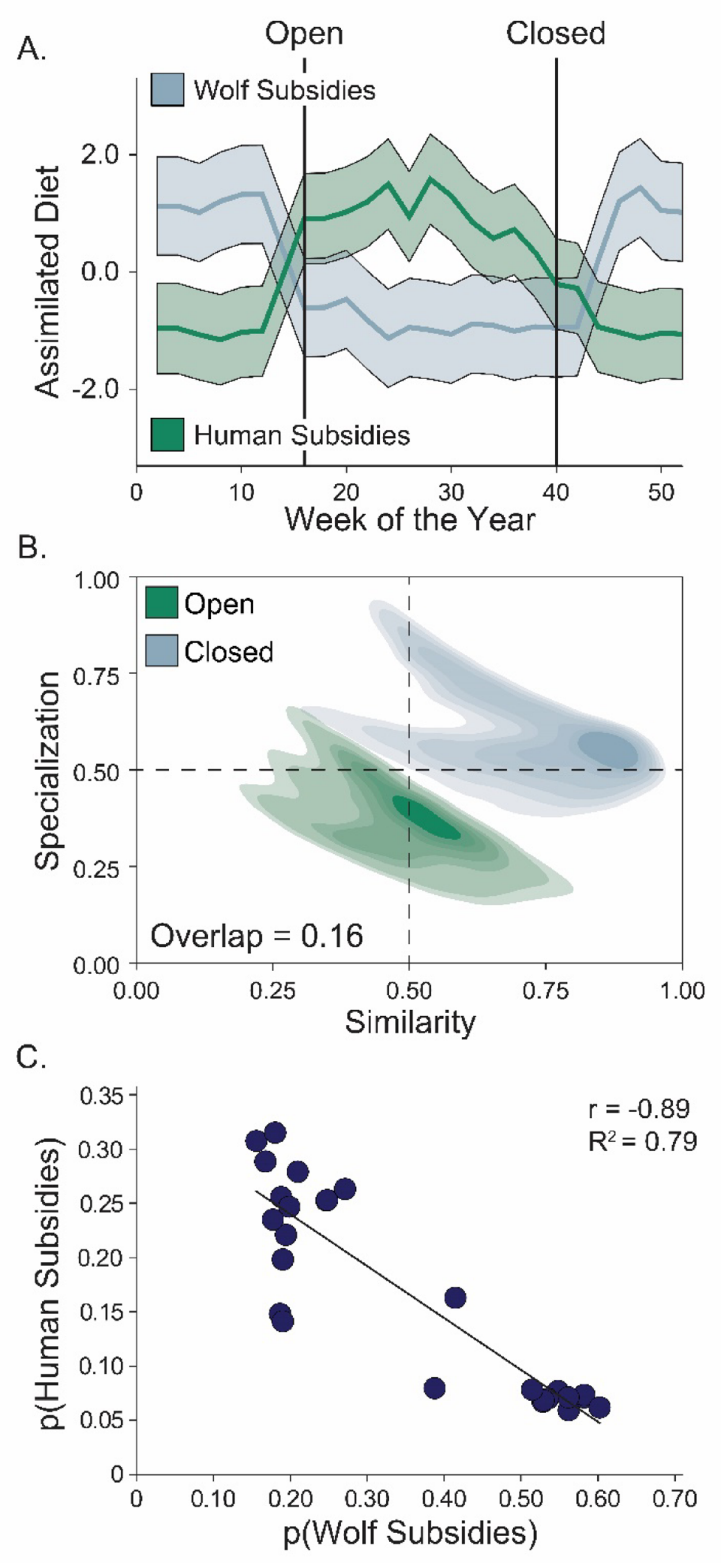
(**A**) Standardized proportion (and 95% confidence intervals) of assimilated resource subsidies from wolves (blue) and humans (green) in the diets of red foxes (*Vulpes vulpes*) based on our individual mixing models using whisker samples; above (**A**), we show the timing of Isle Royale National Park opening and closing to the public. (**B**) Foraging strategies of foxes quantified as similarity (*s*; x-axis) and specialization (µ; y-axis) when the park is open (green) and closed (blue) to visitors. The kernels shown are the 95% confidence kernels (transparent colors) ranging to the core kernels during each season. (**C**) Relationship between the proportion of wolf subsidies and human food subsidies in the diets of red foxes.

Analyzing segmented whiskers to obtain individual fox diet at 2-week intervals, we found that foxes were highly seasonal in their foraging strategies (Colwell’s M/*P* = 0.87 + 0.03, see *SM Results*), consuming large amounts of human food subsidies in summer (0.28 ± 0.02 compared to 0.15 ± 0.05 in winter) and a strong reliance on scavenging of wolf carrion in the winter (0.62 ± 0.04 compared to 0.32 ± 0.04 in summer; Fig. 2B). Corroborating our findings of isotopic niche breadth, the summer diets of foxes – featuring berries (0.24 ± 0.05), small prey (0.16 ± 0.02), and human foods (0.28 ± 0.02) – were more diverse compared to their winter diets, when individuals heavily relied on scavenged carcasses (0.62 ± 0.04) and, to a lesser degree, small prey (0.20 ± 0.02; Fig. 2B and SI Fig. 1). Further, individual dietary overlap was slightly lower in summer (0.57 ± 0.05) than winter (0.63 ± 0.05). Finally, the trophic strategies of foxes exhibited little overlap across seasons (O = 0.10; SI Fig. 4), and their diets were more specialized and similar to one another during winter (µ_Winter_ = 0.57 ± 0.15 and *s*_Winter_ = 0.61 ± 0.20) compared to summer when foxes exhibited more generalized foraging strategies and exhibited higher interindividual variability (µ_Summer_ = 0.38 ± 0.15 and *s*_Summer_ = 0.45 ± 0.18; O_Strategy_ = 0.10; Fig. 3B).

The consumption of human resource subsidies and wolf-killed carrion in fox diets strongly coincided with the timing of the park opening and closing (Fig. 3A). Indeed, within two weeks of park opening, human food consumption increased by ~ 85% and slowly decreased following peak consumption. Meanwhile, wolf subsidies doubled ~ 1 month after the park closed and then decreased by 50% following park opening. Additionally, we detected a strong seasonal signal in fox diets (SI Fig. 4A), and the signs of temporal autocorrelation flipped at park opening and closing (SI Fig. 4B). Given these abrupt shifts in diet, the consumption of human subsidies and wolf subsidies by foxes were strongly negatively correlated (*r* = -0.89, R^2^ = 0.79; Fig. 3C).

## Discussion

Unsurprisingly, the diet of foxes was shaped by resource availability, incorporating diet items as they became available. Specifically, red foxes were trophically facilitated by wolves in winter and then humans in summer. Our findings suggest that the diets of meso-carnivores can be dynamic, and coupled with environmental seasonality and resource availability, likely resulting from long-term adaptations to cope with seasonal changes^22^. These findings also identify a putative mechanism – the asynchronous and complimentary resource subsidies provided by apex carnivores and humans – by which meso-carnivore populations may not be suppressed by large carnivores as would heretofore be anticipated. While Isle Royale is relatively insular – with fewer species and wolf and moose densities being higher compared to mainland systems – this system provided an opportunity to examine how seasonally fluctuating resource availability mediated interspecific interactions, such as scavenging and behavioral responses by meso-carnivores. On its surface, the overall reliance of foxes on human foods was somewhat surprising given that Isle Royale is a relatively pristine wilderness area^40^, where human visitation is both limited in number (25,000–29,000 visitors per year – the least visited National Park in the U.S.), feeding wildlife is prohibited and the park is closed from November to April. However, human use of Isle Royale National Park is confined largely to campgrounds and trails, suggesting foxes are keying in on these features. In environments outside of a park or natural landscape, we would anticipate stronger impacts of human subsidies decoupling foraging strategies of meso-carnivores where there is greater land use and habitat change, as well as human presence.

The summer diets of foxes were more diverse than winter diets and featured a relative uniform reliance on berries, small prey, and human foods. We often detected multiple diet items in the scats of foxes during summer (e.g., berries and mice, insects and squirrels, etc.), though this was not surprising as these diet items are generally small and are all seasonal. On the other hand, the strong reliance of foxes on human foods in the summer was surprising. Wilderness areas were established as “as an area where the earth and its community of life are untrammeled by man, where man himself is a visitor who does not remain” (U.S. Wilderness Act, 1964, pg. 1131–1136, as amended). In contrast, our findings join other research on how expansive and underappreciated direct and indirect human impacts can be^41^, even within protected areas^15^. Nevertheless, the timing of human subsidies aligned closely with the opening and closing of the park (Fig. 3A), and the annual phenological burgeoning resource availability in meso-carnivore summer diets.

Facultative scavengers^22^ exhibit high dietary flexibility^42,43^, as such, red foxes, and other meso-carnivores, can be responsive to resource subsidies in times of scarcity^22^. In Grand Teton National Park, U.S., resource scarcity during winter months led Rocky Mountain red foxes (*Vulpes vulpes macroaura*) to rely on human foods^44^. For comparison, human food consumption was seasonally different in Grand Teton National Park, likely because trophic facilitation was not observed, while the lack of recreators in Isle Royale during winter means foxes must turn to large carnivore subsidies. These findings indicate that foxes incorporate human foods when they are available and reinforce the importance of resource subsidies during times of scarcity. Beyond the seasonal diet on Isle Royale, which features park visitors only during the summer, our results along with others (e.g^44,45^) support the importance of individual and seasonal dietary flexibility (e.g., Fig. 3B) previously observed in meso-carnivores, and further suggest that the resource subsidies that shape community interactions can be independent of the species supplying them.

Foxes consumed ~ 2x more carrion biomass during winter compared to summer (Fig. 2), and we detected moose in ~ 5x more scats during winter (SI Fig. 1), contributing to a higher dietary similarity. Although the degree to which trophic facilitation impacts competitive interactions between carnivores has received widespread attention, few studies have quantified the degree to which seasonal suppression and facilitation can occur. Species that rely on trophic facilitation (i.e., scavenging) are forced to weigh the benefits of a free meal against the risk of intraguild aggression^35,46^. Scavenging draws subordinate carnivores into implicitly risky places for foraging, forcing decisions between food and safety^5^. Given the broad empirical support of the fatal attraction hypothesis^5,35^, our results imply that the impacts of suppression would be highest during winter, the time of the year that subordinate carnivores are mostly reliant on trophic facilitation. Accordingly, winter – or any period of resource scarcity – can represent a bottleneck for carnivores which are often reliant on decreasing resources and, simultaneously, trophic facilitation, which can lead to costly encounters with dominant guild members. While the natural mortality of ungulates is typically highest during winter, and carrion is preserved by cold temperatures, wolf predation introduces carcasses that are more accessible due to the nature of predator kills – often open, partially consumed carcasses, emanating strong olfactory cues that attract scavengers – and conspicuous due to avian scavengers – often accompanied by auditory cues – creating dynamic scavenging opportunities not present when ungulates die of natural causes. To better predict the extent of returning species interactions – particularly as large carnivores are returning to northern latitudes^7^ – we must account for environmental seasonality at the risk of miscalculating the strength of suppression, facilitation or both.

While seasonal subsidies play a central role in community interactions within carnivores guilds^47,48^, the two forms of subsidies that we observed – human foods in summer and wolf-killed carrion in winter – likely have dissimilar impacts on competitive interactions. In more disturbed landscapes, human resource subsidies can alter competitive dynamics and dietary overlap within carnivores^12^. The seasonal human food subsidies on Isle Royale, however, offer a resource that is relatively free of immediate mortality risk. This relatively low-risk and additive resource can lead meso-carnivore populations to increase beyond their natural carrying capacities, which may negatively impact communities in highly seasonal environments. Indeed, factors that alter or inhibit the capacity of consumers to decouple from declining resources would be predicted to destabilize food webs^49^. For example, resource subsidies from humans in the summer could lead to a feedback loop with trophic facilitation from large carnivores during times of resource scarcity whereby meso-carnivores take greater risks to meet their energetic demands during times of resource scarcity due to (i) inflated population sizes, (ii) heightened exploitative competition, or (iii) profuse risk-management. Accordingly, the synchrony of meso-carnivores to a singular dietary item – via changes in resource subsidies provided by humans – could lead to food web instability^49^ by buttressing natural instability during summer. Nevertheless, Rodriguez Curras et al.^15^ did not find a decrease in the fox population as wolves first established on Isle Royale following their reintroduction and after wolf packs coalesced and spatial suppression relaxed. Indeed, while wolves occasionally kill foxes, foxes appear to experience less intense interference competition from wolves than do larger-sized meso-carnivores, like coyotes, and may even benefit indirectly via suppression of dominant competitors. We hypothesize that the benefits that foxes receive from trophic facilitation during winter, compounded with greater resource availability and high levels of human subsidies in summer, likely buffer meso-carnivores from any demographic consequences. Specifically, we attribute the asynchronous and complimentary resource subsidies to the resilience of meso-carnivores on Isle Royale. More broadly, asynchronized resource subsidies provided by humans and large carnivores could strongly influence the diets of meso-carnivores and lead to altered species interactions. Our findings in a relatively undisturbed landscape limited to non-consumptive quiet recreation may help explain why we are not seeing consistent meso-carnivore suppression elsewhere, particularly in landscapes featuring high levels of human resource subsidies.

## Conclusion

Understanding seasonal variations in resource use is important for predicting how species interactions cascade through carnivore communities^22^. Globally, large carnivores are returning to their historical communities^7^ and reintroducing large carnivores is a widely adopted strategy to restore ecological interactions^6^. As large carnivores return, the resource subsidies they provide to obligate and facultative scavengers is predicted restore community stability. Due to the disproportionate impacts that carnivores have on community- to ecosystem-level processes^50^, restoring the functional role of carnivores has been classified as a priority for conservation^51^. On Isle Royale, the reintroduction of wolves restored resource subsidies to foxes^15^, which we show are an importantcomponent of fox diet during winter (e.g., Figs. 2 and 3; SI Fig. 1). Resource subsides can stabilize community interactions^52^, and our results suggest these anticipated interactions have, likely, been restored. Alternatively, human resource subsidies associated with summer recreation – even in one of the least visited National Parks in the United States – created a resource subsidy that is relatively free of risk, altering the trophic ecology of this carnivore. The impacts from seasonally asynchronized human and large carnivore resource subsidies likely play an underappreciated role in structuring contemporary carnivore communities, may upend meso-predator suppression, and contribute to the “rise of the meso-predator”.

## Methods

### Study area

Isle Royale (544 km2) is in north-western Lake Superior, USA (48°N, 89°W). The island exhibits a transition deciduous-to-boreal forest. Mean monthly seasonal temperatures range from − 9 °C in January to 15.8 °C in July, while mean annual precipitation is 75 cm, ranging from 54 cm to 107 cm with 40% of annual precipitation falling as snow. The current terrestrial carnivore community on Isle Royale is relatively simple: wolves, red foxes, and American martens. The other carnivores on the island are mostly or entirely aquatic, including American mink (*Neogale vison*) and North American river otter (*Lontra canadensis*), or a occur at very low densities (i.e., ermine [*Mustela erminea*]).

Wolves were reintroduced to Isle Royale in 2018 to manage the parks increasing moose (*Alces alces*) population. Immediately following their reintroduction, wolves had an initially strong effect on the red fox distribution and space use – foxes were more likely to be found near human campgrounds and detected in higher human-use areas – but these effects quickly subsided and there were no persisting effects to the fox population^15^. Isle Royale is closed to public access from September 15 – April 15, limiting potential virtually all resource subsides from humans to the summer months. Alternatively, carrion availability increases during winter due to high rates of winter mortality though its access is largely mediated by wolves.

### Stable isotope data collection and Preparation

From September 2021 through February 2024, we captured 16 red foxes using foothold traps, box traps, and cable restraints (N_Winter_=8 and N_Summer_=8) and whisker samples were collected for stable isotope analysis. The fox population in our study area was ~ 45 individuals^15^, so we effectively sampled ~ 1/3 of the population during our study period. During the summer and winters, we also collected 442 scats (378 in Summer and 64 in Winter; SI Table 1) along the major trail system in Isle Royale and analyzed scat to inform our stable isotope analysis (see below). All protocols were approved by the Institutional Animal Care and Use Committee at the University of Wisconsin-Madison and the National Park Service (A006483).

Red fox whiskers grow at a constant rate^53,54^ of 0.43 ± 0.10 mm/day^55^. Accordingly, we segmented whiskers to a length of 7 mm to balance the weight required for accurate bulk stable isotope analysis (0.5–0.6 mg) and sufficient temporal resolution to reconstruct diets across seasons; each sample, then, represented ~ 18 days. In total, we sampled 88 whisker segments, representing 5.5 ± 1.6 segments per individual fox (SI Table 3). Whisker samples were rinsed three times with 2:1 chloroform: methanol solution, segmented and homogenized with surgical scissors, and then dried at 55 °C for a minimum of 72 h following previously described methods^56^. Because whiskers (and fur samples for our prey in our past study) are keratinous, we did not lipid extract these samples.

We measured the carbon (δ^13^C) and nitrogen (δ^15^N) isotope values were measured using a Costech 4010 Elemental Analyzer (Valencia, CA) coupled to a Thermo Scientific Delta V Plus (Bremen, Germany) isotope ratio mass spectrometer with internal reference materials calibrated against international reference standards (V-PDB for δ^13^C and atmospheric N for δ^15^N). The within-run standard deviation for internal reference materials was < 0.2‰ for both δ^13^C and δ^15^N values. All measurements were conducted at the University of California-Davis (Davis, CA, USA).

### Stable isotope data analyses

To test the mechanisms driving variation in diet seasonally – at both the population- and individual-level – we used δ^13^C and δ^15^N values to estimate the isotopic niche breadth and isotopic niche overlap in ‘δ-space’^57,57,59^ and the proportional assimilation of putative prey and dietary overlap based on stable isotope mixing models (i.e., ‘p-space’^60^. First, we used an ANOVA to test the differences in δ^13^C and δ^15^N at the population level across seasons. We quantified isotopic niche breadth within seasons and overlap across seasons at the population-level – a proxy for dietary niche breadth and competition, respectively – using the 95% standard ellipses areas corrected for small sample sizes (SEAcs) in the R package *SIBER v 2.1.6*^60^. We ran 3 chains of 300,000 iterations and removed the first 200,000 iterations as burn-in and then thinned posterior samples to every 10th sample. To quantify isotopic niche overlap, we used the single metric of niche overlap (O_δ_) defined by overlap as the proportion of non-overlapping area of the two ellipses relative to the non-overlapping area, $\:{O}_{\delta\:}={O}_{W-S}/\left(\left({B}_{W}+{B}_{S}\right)-{O}_{W-S}\right)$, where *O*_*S−W*_ (= *O*_*W−S*_) is the overlap across seasons, *B*_*W*_ is the niche breadth of foxes in winter and *B*_*S*_ is the isotopic niche breadth during summer; the resulting value ranges from 0 (no overlap) to 1.0 (complete overlap).

To determine the proportional assimilation of dietary groups of foxes across seasons we used concentration-dependent mixing models using the R package *simmr 0.4.5*^62^. For all prey endmembers, we used previously collected^15^ dietary samples analyzed for bulk δ^13^C and δ^15^N. To account for potential human resource subsidies in carnivore diets, we also included isotopic ratios from anthropogenic sources, including human refuse and domestic prey^63,64^. We included berries that would be available during the hair growth period during the summer only (i.e., spring-early-summer^65^; and aggregated isotopically indistinct prey items, using a k-means clustering algorithm^66^ in the R package *NbClust v3.0.1*^67^. We identified 4 isotopically distinct and biologically meaningful prey groups representative of Isle Royale: (1) berries (e.g., Rubus spp. and Fragaria spp.), (2) wolf carrion (moose and snowshoe hares [Lepus americanus] – this isotopic group was principally moose, which was predominantly obtained through scavenging wolf kills, so we refer to it as wolf carrion), (3) small prey (e.g., beaver [*Castor canadensis*], mice [*Peromyscus maniculatus*], red squirrels [*Tamiascirus hudsonicus*], Arthropods, and birds [e.g., *Leuconotopicus villosus* and *Dendroica spp.*]), and (4) human foods. Putative prey samples included: moose (*N* = 17), beaver (*N* = 5), snowshoe hare (*N* = 7), red squirrel (*N* = 5), deer mice (*N* = 7), passerine birds (*N* = 15), arthropods (*N* = 5), and berries (*N* = 23; see SI Fig. 2).

We estimated proportional diet at the population- and individual-level by running 3 chains of 300,000 iterations and removed the first 200,000 iterations as burn-in and then thinned posterior samples to every 10th sample. We used [C] and [N] data of berries ([C] = 0.48; [N] = 0.01), wolf carrion ([C] = 0.47; [N] = 0.14), and small prey ([C] = 0.47; [N] = 0.14) from Carlson et al.^68^, and data from Hopkins and Ferguson^69^ for the concentrations of anthropogenic-derived sources ([C] = 0.53; [N] = 0.07). Lastly, we used diet-specific trophic discrimination factors of Δ^13^C=2.5 and Δ^15^*N*=3.4 for all hair and whisker samples^70,71^ except for human foods. Because we sampled potential prey and wolf carrion (moose) hair, we adjusted our Δ^13^C discrimination factors by − 1.0‰ for those prey groups to account for tissue specific discrimination factors^64,70^. We used trophic discrimination factors of Δ^13^C=2.0 and Δ^15^*N*=3.5 for human foods following Newsome et al.^72^.

To estimate the proportions of each diet item, we used informative priors based on our previous work in Isle Royale^15,73^, the known number of visitors to Isle Royale throughout the year, and our scat analysis from summer and winter to inform our model. First, we used uniform priors (i.e., even dietary proportions, i.e., 1/N-sources = 0.25) throughout the modelling process^60,74^ to test the assimilation of dietary sources without our prior information for baseline estimates from the mixing space (SI Fig. 3). As expected, summer values estimated higher proportions of moose and lower proportions of small prey and human foods based on evidence from our scat analysis and previous research on Isle Royale. Further, during winter, our uniform mixing model over-estimated the proportion of human foods and under-estimated the proportions of small prey. To include prior information from our scat analysis (see SM Methods and Results; SI Fig. 1) and previous research on Isle Royale^15,73^, we used standardized priors of 0.17 ± 0.18, -0.69 ± 0.30, 0.52 ± 0.05, and 0.001 ± 0.22 during summer. For our winter estimates, we accounted for the park closure between September 15 and April 15 and the broadly known phenology of fruiting vegetation and used standardized priors of 0.69 ± 0.04, 1.82 ± 0.04, -1.58 ± 0.39, and − 0.94 ± 0.50 for small prey, wolf carrion, human foods, and berries, respectively.

We first quantified prey use at the population level by grouping each individual within summer and winter (with individual as a random effect) and estimated proportional use of the four potential prey sources (berries, wolf carrion, small prey, and human foods). Then, we analyzed individual whisker segments separately to quantify consumption of each prey group at each represented window (~ 18 days) and tested the impacts of individuals on dietary variability through the seasons. For our individual-level analyses, we included the mean priors, but left the standard deviations uninformed so that the models would capture the individual variability around the means. For each analysis, we identified model convergence using the Gelman–Rubin diagnostic value R̂ < 1.01 and the effective sample size of each estimate > 5000. Because our estimates of dietary proportions are posterior distributions, we calculated the proportion of overlap between posterior distributions. We estimated dietary niche breadth and overlap using our posterior dietary proportion estimates of individuals across seasons in the R package *nicheROVER v.1.9.0*^75^.

To quantify the foraging strategies of individuals within each season, we used the individual proportional estimates of prey groups to quantify dietary specialization (*ε*) and similarity (*s*) indices and visually inspected the density plots^61^. Here, *ε* varies between 0 (an ultra-generalist) and 1 (an ultra-specialist) and *s* varies between 0 (exactly dissimilar from the population) and 1 (exactly similar to the population). Accordingly, the density plots can be subdivided into four quadrants: dietary specialists (*ε* > 0.50) with diets dissimilar to the population (*s* < 0.50; dissimilar-specialists), dietary specialists with diets similar to the population (s > 0.50; similar-specialists), dietary generalists (*ε*< 0.50) with diets dissimilar to the population (dissimilar-generalists), and dietary generalists with diets similar to the population (similar-generalists;^61^. We classified dietary specialization and similarity from the posterior estimates of individual diets and generated density plots to classify the foraging strategies at the population level based on where the density distributions using a kernel function (Newsome et al. 2012) – foraging strategies are qualified as unique if there is little overlap between the density kernels.

We estimated dietary overlap across seasons using Pinaka’s index of niche overlap^76^ in the R package pgirmess v2.0.3^77^ using a bootstrap estimate of *N* = 10,000. We also used a non-parametric test to test for differences in diet use across seasons in the R package *npvm v2.4.0*^78^. Finally, we used a similarity percentage analysis in the R package *vegan v2.6-2*^79^ to determine percent contribution of each prey group to the dissimilarity in diet composition among groups based on a Bray–Curtis dissimilarity matrix calculated from the estimates of each of prey group.

To test for seasonality using our stable isotope data and the consumption of wolf carrion and human foods from our mixing model results, we tested for contingency (Colwell’s M;^80^, the standardized role of seasonality in relation to overall predictability (Contingency/ Contingency + Constancy; M/C + M), and a wavelet analysis to independently identify dominant temporal cycles and characterize the predictability of seasonal red fox diet; though we initially tested for temporal autocorrelation between summer and winter in the base stats package in R^81^. From a standard definition of seasonal predictability – the regularity of recurrence of the annual distribution of events – we quantified predictability as the proportion of timesteps over the time series with significant power at the 26-week frequency (i.e., two-week intervals in general accordance with the timing of stable isotope data collected from individual whiskers; each whisker segment represents an average of 18 days of assimilated data). First, we combined our stable isotope data from the 16 captured individuals across three years into a single dataset using the Julian date of each capture. We assigned the d^15^N, wolf carrion, and human food data from each whisker segment (*N* = 88) over the 18-days of the year that each segment represented. We then took a running two-week average of each segment that fell into the desired time window to capture the seasonal repeatability of the red fox diet. To calculate contingency and constancy, we used the R package *hydrostats v.0.2.9*^82^. To perform the wavelet analysis, we used the R package *WaveletComp v.1.1*^83^. We used the Morlet wavelet which represents a sine wave modulated by a Gaussian function. Finally, we used a time series analysis in the R package *MARSS v.3.11.9*^84^ using a simplified parameter matrix to minimize model complexity and capture the broad yearly pattern in our data. Testing for seasonality from time-series data does not use any prior categorization (i.e., pre-defined, subjective seasonal characterization) to inform the analysis, so it is a powerful tool to test for periodicity in time-series data, which has not been previously applied to trophic ecology. All analyses were performed in *R*^81^.

## Supplementary Information

Below is the link to the electronic supplementary material.

Supplementary Material 1

## Data Availability

All data used in this manuscript is available through FigShare ( [https://doi.org/10.6084/m9.figshare.27957498.v2](https:/doi.org/10.6084/m9.figshare.27957498.v2) ).

## References

[1] Ripple, W. J. et al. Status and ecological effects of the world’s largest carnivores. *Science ***343**(6167):1241484 (2014). 10.1126/science.1241484 24408439

[2] Crooks, K. R. & Soule, M. E. Mesopredator release and aviafauna extinctions in a fragmented landscape. *Nature* **400**, 563–566 (1999).

[3] Donadio, E. & Buskirk, S. W. Diet, morphology, and interspecific killing in carnivora. *Am. Nat.* **167**, 524–536 (2006). 16670995 10.1086/501033

[4] de Oliveira, T. G. & Pereira, J. A. Intraguild predation and interspecific killing as structuring forces of carnivoran communities in South America. *J. Mamm. Evol.* **21**, 427–436 (2014).

[5] Prugh, L. R. & Sivy, K. J. Enemies with benefits: integrating positive and negative interactions among terrestrial carnivores. *Ecol. Lett.* **23**, 902–918 (2020). 32185877 10.1111/ele.13489

[6] Wolf, C. & Ripple, W. J. Rewilding the world ’s large carnivores. *R. Soc. Open. Sci.* **5**, 172235 (2018). 10.1098/rsos.180514 PMC5990845 29893379

[7] Chapron, G. et al. Recovery of large carnivores in Europe’s modern human-dominated landscapes. *Science ***346**, 1517-1519 (2014). 10.1126/science.1257553 25525247

[8] Prugh, L. R. et al. The rise of the mesopredator. *Bioscience* **59**, 779–791 (2009).

[9] Venter, O. et al. Sixteen years of change in the global terrestrial human footprint and implications for biodiversity conservation. *Nat. Commun.* **7**, 1–11 (2016). 10.1038/ncomms12558 PMC4996975 27552116

[10] Schug, F. et al. The global wildland–urban interface. *Nature* **621**, 94–99 (2023). 37468636 10.1038/s41586-023-06320-0 PMC10482693

[11] McKinney, M. L. & Lockwood, J. L. Biotic homogenization: A few winners replacing many losers in the next mass extinction. *Trends Ecol. Evol.* **14**, 450–453 (1999). 10511724 10.1016/s0169-5347(99)01679-1

[12] Manlick, P. J. & Pauli, J. N. Human disturbance increases trophic niche overlap in terrestrial carnivore communities, *Proc. Natl. Acad. Sci.* **117(**43), 26842-26848 (2020). 10.1073/pnas.2012774117 PMC7604423 33046630

[13] Kuijper, D. P. J. et al. Paws without claws? Ecological effects of large carnivores in anthropogenic landscapes. *Proceedings of the Royal Society B: Biological Sciences* 283, (2016). 10.1098/rspb.2016.1625 PMC5095381 27798302

[14] van Schaik, T., van Kuijk, M. & Sterck, E. H. M. Understanding mesopredator responses to changes in apex predator populations in europe: implications for the mesopredator release hypothesis. *Mamm. Rev.* **1–15**10.1111/mam.12357 (2024).

[15] Rodriguez Curras, M., Romanski, M. C. & Pauli, J. N. The pulsed effects of reintroducing wolves on the carnivore community of Isle Royale. *Front. Ecol. Environ.* **22**, 1–7 (2024).

[16] Perrig, P. L., Donadio, E., Middleton, A. D. & Pauli, J. N. Puma predation subsidizes an obligate scavenger in the high Andes. *J. Appl. Ecol.* **54**, 846–853 (2017).

[17] Wilson, E. E. & Wolkovich, E. M. Scavenging: how carnivores and carrion structure communities. *Trends Ecol. Evol.* **26**, 129–135 (2011). 21295371 10.1016/j.tree.2010.12.011

[18] Selva, N. & Fortuna, M. A. The nested structure of a scavenger community. *Proc. Royal Soc. B: Biol. Sci.* **274**, 1101–1108 (2007). 10.1098/rspb.2006.0232 PMC2124470 17301021

[19] Yang, L. H., Bastow, J. L. & Spence, K. O. & Wright, A. N. What can we learn from resource pulses? *Ecology* 89, 621–634 (2008). 10.1890/07-0175.1 18459327

[20] Berger, K. M., Gese, E. M. & Berger, J. Indirect effects and traditional trophic cascades : A test involving wolves, coyotes, and pronghorn. *Ecology* **89**, 818–828 (2008). 18459344 10.1890/07-0193.1

[21] Deacy, W. W. et al. Phenological synchronization disrupts trophic interactions between Kodiak brown bears and salmon. *Proc. Natl. Acad. Sci. U S A*. **114**, 10432–10437 (2017). 28827339 10.1073/pnas.1705248114 PMC5625906

[22] Pereira, L. M., Owen-Smith, N. & Moleón, M. Facultative predation and scavenging by mammalian carnivores: Seasonal, regional and intra-guild comparisons. *Mammal Review* vol. 44 44–55 Preprint at (2014). 10.1111/mam.12005

[23] Osterback, A. M. K., Frechette, D. M., Hayes, S. A., Shaffer, S. A. & Moore, J. W. Long-term shifts in anthropogenic subsidies to gulls and implications for an imperiled fish. *Biol. Conserv.* **191**, 606–613 (2015).

[24] Abraham, A. J., Doughty, C. E., Plummer, K. E. & Duvall, E. S. Supplementary bird feeding as an overlooked contribution to local phosphorus cycles. *Front. Ecol. Environ.* **1–7**10.1002/fee.2793 (2024).

[25] Moss, W. E., Alldredge, M. W., Logan, K. A. & Pauli, J. N. Human expansion precipitates niche expansion for an opportunistic apex predator (Puma concolor). *Sci. Rep.* **6**, 2–6 (2016). 28008961 10.1038/srep39639 PMC5180354

[26] Kirby, R., Alldredge, M. W. & Pauli, J. N. The diet of black bears tracks the human footprint across a rapidly developing landscape. *Biol. Conserv.* **200**, 51–59 (2016).

[27] Murray, M., Edwards, M. A. & Abercrombie, B. & St. Clair, C. C. Poor health is associated with use of anthropogenic resources in an urban carnivore. *Proceedings of the Royal Society B: Biological Sciences* 282, (2015). 10.1098/rspb.2015.0009 PMC4426620 25876843

[28] Pluemer, M. et al. Red foxes (Vulpes vulpes) and Coyotes (Canis latrans) in an urban landscape: prevalence and risk factors for disease. *J. Urban Ecol.* **5**, 1–9 (2019).

[29] Brauman, K. A. et al. Global trends in nature’s contributions to people. *Proc. Natl. Acad. Sci. U S A*. **117**, 32799–32805 (2020). 33288690 10.1073/pnas.2010473117 PMC7768808

[30] Moll, R. J. et al. Humans and urban development mediate the sympatry of competing carnivores. *Urban Ecosyst.* **21**, 765–778 (2018).

[31] Mueller, M. A., Drake, D. & Allen, M. L. Coexistence of Coyotes (Canis latrans) and red foxes (Vulpes vulpes) in an urban landscape. *PLoS One*. **13**, 1–19 (2018). 10.1371/journal.pone.0190971 PMC5783369 29364916

[32] Wilmers, C. C., Stahler, D. R., Crabtree, R. L., Smith, D. W. & Getz, W. M. Resource dispersion and consumer dominance: scavenging at wolf- and hunter-killed carcasses in greater yellowstone, USA. *Ecol. Lett.* **6**, 996–1003 (2003).

[33] Sivy, K. J., Pozzanghera, C. B., Grace, J. B. & Prugh, L. R. Fatal attraction? Intraguild facilitation and suppression among predators. *Am. Nat.* **190**, 663–679 (2017). 29053355 10.1086/693996

[34] Prugh, L. R. et al. Fear of large carnivores amplifies human-caused mortality for mesopredators. *Science* **380**, 754–758 (2023). 37200434 10.1126/science.adf2472

[35] Ruprecht, J. et al. Variable strategies to solve risk-reward tradeoffs in carnivore communities. *Proc. Natl. Acad. Sci. U S A*. **118**, 1–9 (2021). 10.1073/pnas.2101614118 PMC8536332 34429359

[36] Mech, L. et al. An unparalleled opportunity for an important ecological study. *Bioscience* **67**, 875–876 (2017).

[37] Hedrick, P. W., Robinson, J. A., Peterson, R. O. & Vucetich, J. A. Genetics and extinction and the example of Isle Royale wolves. *Anim. Conserv.* **22**, 302–309 (2019).

[38] Mark, C. et al. Wolves and the Isle Royale Environment: Restoring an Island Ecosystem (2018). https://wolfwatcher.org/wp-content/uploads/2020/10/NPS-SUNY-Isle-Royale-Wolf-Summary-Report-2018-2020.pdf (2020).

[39] Hoy, S. R., Vucetich, L. M., Peterson, R. O. & Vucetich, J. A. Winter tick burdens for moose are positively associated with warmer summers and higher predation rates. *Front Ecol. Evol* (9), (2021).

[40] Radeloff, V. C. et al. The rise of novelty in ecosystems. *Ecol. Appl.* **25**, 2051–2068 (2015). 26910939 10.1890/14-1781.1

[41] Sarmento, W. M. & Berger, J. Human visitation limits the utility of protected areas as ecological baselines. *Biol. Conserv.* **212**, 316–326 (2017).

[42] Johnson, W. J. Food habits of the red Fox in Isle Royale National park. *Am. Midl. Nat.* **84**, 568–572 (1970).

[43] Scholz, C. et al. Individual dietary specialization in a generalist predator: A stable isotope analysis of urban and rural red foxes. *Ecol. Evol.* **10**, 8855–8870 (2020). 32884662 10.1002/ece3.6584 PMC7452770

[44] Burkholder, E. N. et al. How does anthropogenic food influence the trophic ecology of Rocky mountain red fox?? *J. Mammal*. 10.1093/jmammal/gyae108 (2024).

[45] Bolnick, D. I. et al. The ecology of individuals: incidence and implications of individual specialization. *Am. Nat.* **161**, 1–28 (2003). 12650459 10.1086/343878

[46] Lima, S. L. & Bednekoff, P. A. Temporal variation in danger drives antipredator behavior: the predation risk allocation hypothesis. *Am. Nat.* **153**, 649–659 (1999). 29585647 10.1086/303202

[47] Polis, G. A. & Strong, D. R. Food web complexity and community dynamics. *Am. Nat.* **147**, 813–846 (1996).

[48] Huxel, G. R. & McCann, K. Food web stability: the influence of trophic flows across habitats. *Am. Nat.* **152**, 460–469 (1998). 18811452 10.1086/286182

[49] McMeans, B. C., McCann, K. S., Humphries, M., Rooney, N. & Fisk, A. T. Food web structure in Temporally-Forced ecosystems. *Trends Ecol. Evol.* **30**, 662–672 (2015). 26452520 10.1016/j.tree.2015.09.001

[50] Ritchie, E. G. et al. Ecosystem restoration with teeth: what role for predators? *Trends Ecol. Evol.* **27**, 265–271 (2012). 22321653 10.1016/j.tree.2012.01.001

[51] *Rewilding (Ecological Reviews)*. (Cambridge University Press, Cambridge, (2019). 10.1017/9781108560962

[52] Polis, G. A. & Hurd, S. D. *Allochthonous Inputs across Habitats, Subsidized Consumers, and Apparent Trophic Cascades: Examples from the Ocean-Land Interface* (Springer, 1995).

[53] Robertson, A., McDonald, R. A., Delahay, R. J., Kelly, S. D. & Bearhop, S. Whisker growth in wild Eurasian badgers Meles meles: implications for stable isotope and bait marking studies. *Eur. J. Wildl. Res.* **59**, 341–350 (2013).

[54] Mutirwara, R., Radloff, F. G. T. & Codron, D. Growth rate and stable carbon and nitrogen isotope trophic discrimination factors of Lion and Leopard whiskers. *Rapid Commun. Mass Spectrom.* **32**, 33–47 (2018). 28971533 10.1002/rcm.8003

[55] McLaren, A. A. D., Crawshaw, G. J. & Patterson, B. R. Carbon and nitrogen discrimination factors of wolves and accuracy of diet inferences using stable isotope analysis. *Wildl. Soc. Bull.* **39**, 788–796 (2015).

[56] Pauli, J. N., Ben-David, M., Buskirk, S. W., Depue, J. E. & Smith, W. P. An isotopic technique to mark mid-sized vertebrates non-invasively. *J. Zool.* **278**, 141–148 (2009).

[57] Layman, C. A., Arrington, D. A., Montaña, C. G. & Post, D. M. Can stable isotope ratios provide for community-wide measures of trophic structure? *Ecology* **88**, 42–48 (2007). 17489452 10.1890/0012-9658(2007)88[42:csirpf]2.0.co;2

[58] Newsome, S. D., Rio, C. M., del, Bearhop, S. & Phillips, D. L. A niche for isotopic ecology. *Front. Ecol. Environ.* **5**, 429–436 (2007).

[59] Jackson, A. L., Inger, R., Parnell, A. C. & Bearhop, S. Comparing isotopic niche widths among and within communities: SIBER – Stable isotope bayesian ellipses in R. *J. Anim. Ecol.*10.1111/j.1365-2656.2011.01806.x (2011). 21401589 10.1111/j.1365-2656.2011.01806.x

[60] Parnell, A. C., Inger, R., Bearhop, S. & Jackson, A. L. Source partitioning using stable isotopes: coping with too much variation. *PLoS One*. **5**, 1–5 (2010). 10.1371/journal.pone.0009672 PMC2837382 20300637

[61] Newsome, S. D., Yeakel, J. D., Wheatley, P. V. & Tinker, M. T. Tools for quantifying isotopic niche space and dietary variation at the individual and population level. *J. Mammal*. **93**, 329–341 (2012).

[62] Parnell, A. C. simmr: A Stable Isotope Mixing Model. *R package version 0.4.6.9000* (2019).

[63] Hülsemann, F. et al. Global Spatial distributions of nitrogen and carbon stable isotope ratios of modern human hair. *Rapid Commun. Mass Spectrom.* **29**, 2111–2121 (2015). 26467223 10.1002/rcm.7370

[64] Newsome, S. D. et al. Individual variation in anthropogenic resource use in an urban carnivore. *Oecologia* **178**, 115–128 (2015). 25669449 10.1007/s00442-014-3205-2

[65] Korhonen, H., Harri, M. & Asikainen, J. Moulting and seasonal pelage variations in the raccoon dog. *Acta Theriol. (Warsz)*. **29**, 77–88 (1984).

[66] Phillips, D. L. et al. Best practices for use of stable isotope mixing models in food-web studies. *Can. J. Zool.* **92**, 823–835 (2014).

[67] Charrad, M., Ghazzali, N., Boiteau, V., Niknafs, A. & Nbclust An R package for determining the relevant number of clusters in a data set. *J. Stat. Softw.* **61**, 1–36 (2014).

[68] Carlson, J. E. et al. Potential role of prey in the recovery of American Martens to Wisconsin. *J. Wildl. Manage.* **78**, 1499–1504 (2014).

[69] Hopkins, J. B. & Ferguson, J. M. Estimating the diets of animals using stable isotopes and a comprehensive bayesian mixing model. *PLoS One ***7**(1): e28478 (2012). 10.1371/journal.pone.0028478 PMC3250396 22235246

[70] Roth, J. D. & Hobson, K. A. Stable carbon and nitrogen isotopic fractionation between diet and tissue of captive red fox: implications for dietary reconstruction. *Can. J. Zool.* **78**, 848–852 (2000).

[71] Stephens, R. B., Ouimette, A. P., Hobbie, E. A. & Rowe, R. J. Reevaluating trophic discrimination factors (∆δ13C and ∆δ15N) for diet reconstruction. *Ecol. Monogr.* **92**, 1–20 (2022).

[72] Newsome, S. D., Garbe, H. M., Wilson, E. C. & Gehrt, S. D. Individual variation in anthropogenic resource use in an urban carnivore. *Oecologia* **178**, 115–128 (2015). 25669449 10.1007/s00442-014-3205-2

[73] Lacin Alas, B. et al. The repatriation of wolves to Isle Royale alters the foraging of meso-carnivores. *J. Mammal*. **106**, 30–38 (2025).

[74] Stock, B. C. et al. Analyzing mixing systems using a new generation of Bayesian tracer mixing models. *PeerJ* 1–27 (2018). (2018). 10.7717/peerj.5096 PMC6015753 29942712

[75] Swanson, H. K. et al. A new probabilistic method for quantifying n-dimensional ecological niches and niche overlap. *Ecology* **96**, 318–324 (2015). 26240852 10.1890/14-0235.1

[76] Pianka, E. R. Niche overlap and diffuse competition (desert lizards/resource partitioning/community structure/species diversity). *Ecology* **71**, 2141–2145 (1974). 10.1073/pnas.71.5.2141 PMC388403 4525324

[77] Giraudoux, P. Package ‘ pgirmess ’. Preprint at (2024).

[78] Ellis, A. R., Burchett, W. W., Harrar, S. W. & Bathke, A. C. Nonparametric inference for multivariate data: the R package Npmv. *J Stat. Softw* **76**(4), 1-18 (2017).

[79] Oksanen, J. et al. vegan: Community Ecology Package. *R package version 2.6-2.* (2022).

[80] Colwell, R. K. Predictability, constancy, and contingency of periodic phenomena. *Ecology* **55**, 1148–1153 (1974).

[81] Team, R. C. R: A language and environment for statistical computing. *R Foundation for Statistical Computing, Vienna, Austria* (2024).

[82] Bond, N. Package ‘hydrostats’: Hydologic indices for daily time series data. R package version 0.2.9. (2022).

[83] Rösch, A., Schmidbauer, H. & WaveletComp Computational Wavelet Analysis. R package version 1.1. 1–38 (2018).

[84] Holmes, E. E., Ward, E. J., Sheuerell, M. D. & Wills, K. MARSS: Multivariate autoregrassive state-space modeling. R package version 3.11.9. (2024).

