# Supplementary material for: Carrion from large carnivores and food from humans subsidize mesocarnivores year round
Source: Sci Rep. 2025 Aug 14;15:29813. doi: 10.1038/s41598-025-15503-w (PMC12354756; doi:10.1038/s41598-025-15503-w)
Supplement: Supplementary file 1 — Supplementary Material 1 [file 41598_2025_15503_MOESM1_ESM.docx]

**Supporting Information for: Carrion from large carnivores and food from humans subsidize meso-carnivores year-round**

**SI Methods**

Scat Data Collection and Preparation

Beginning in late-August through late-September and mid-February through early-March, we hiked the four major trails on the western-end of the island (the Feldtmann [24km], Huginnin [20km], Greenstone [22km], and Minong [18km]) and the Windigo area. During summer, we surveyed each trail a total of seven times, with a four-day interval between sampling efforts, while we sampled each trail three times, with a four-day interval during winter. When a scat was encountered, we swabbed the exterior of the scat with a sterile cotton applicator (Thermo Fisher Scientific, USA) and placed the applicator in a 15ml Falcon tube (Thermo Fisher Scientific, USA) filled with desiccant. We collected the scat sample in a 50ml tube, recorded the location (UTM), and stored frozen within 8 hours of collection. Genetic species identity was accomplished following Lacin-Alas et al. (2024). Briefly, we extracted DNA from all scat samples using Qiagen QIAamp DNA micro kits (QIAGEN, Valencia, CA) in a dedicated pre-PCR room and included a negative control for each extraction batch. We identified samples to species from a species-specific portion of mitochondrial DNA (mtDNA).

We rinsed (using a detergent; Alconax Inc. NY, USA), dried at 65°C overnight (~ 12 hours), and filtered (through a fine mesh) each scat sample prior to analysis. We used a dissecting and light microscope to identify the undigested material to the finest possible taxonomic level (except for birds and arthropods) using keratinous or bone fragments (i.e., skulls, mandibles, teeth, and claws), guard hair coloration, banding patterns, and medulla characteristics and comparing samples to a reference voucher specimen library [1]. We recorded the presence-absence (i.e., occurrence) of each species or item (e.g., seeds and plant material). Accordingly, the sum of occurrences exceeded 100% because we frequently found more than one food item in each scat.

Scat Data Analysis

We used all scat samples collected generate a naïve frequency of occurrence of each diet item [1]. In addition, we also used a bootstrap sample within each season to account for within-individual pseudo-replication. We resampled the dataset N = 10,000 times using each draw to calculate the frequency of occurrence to capture variation of diet at the population level. We further quantified Hill’s diversity and evenness metrics [2] to test changes in diet use across seasons in the R package *chemodiv v0.3.0* [3]. To compare diversity and evenness across seasons, we calculated the total density overlap of the bootstrap draws in the R package *overlapping v.2.1* [4].

**SI Results**

*Summer and Winter Diet (Scat)*

During winter, foxes consumed significantly higher proportions of moose (O<0.001) and snowshoe hares (O = 0.001±0.0006), while in summer, they consumed significantly higher proportions of squirrels (O = 0.004±0.002), birds (O<0.001), arthropods (O<0.001), and berries (O<0.001; Figure 1A). Fox diet was more diverse in summer (6.29±0.14) than winter (4.31±0.31; O<0.001; Figure 1B). Lastly, fox winter (0.75±0.07) and summer (0.70±0.02) diets were similarly evenly distributed (O=0.37±0.01, given the resources available (i.e., no insects or berries were consumed during winter; Figure 1C). Dietary overlap between summer and winter was relatively low (0.30±0.02) and diets across seasons were significantly different (non-parametric bootstrap test p<0.01). The leading causes of dietary dissimilarity across seasons (accounting for the cumulative sum in dissimilarity ≥ 0.75) for foxes was in the consumption of berries, arthropods, moose, and, lastly, birds (SM Table 1).

*Dietary Seasonality*

Using δ^15^N as a proxy for seasonal diets (Figure SM 5A and B), we detected strong positive autocorrelation throughout winter and negative autocorrelation between summer and winter (Figure 5C). Yearly Colwell’s predictability (P; 0.89±0.02) and contingency (M; 0.77±0.02) were both high, while constancy (C) was low (0.13±0.03). Furthermore, the contribution that seasonality contributed to predictability (M/M+C) was high 0.87±0.03 (SM Figure 5A). Similarly, we estimated a strong seasonal impact (M/P; 0.93±0.02; SM Figure 5D).

[1] J. E. Carlson, J. H. Gilbert, J. W. Pokallus, P. J. Manlick, W. E. Moss, and J. N. Pauli, ‘Potential role of prey in the recovery of American martens to Wisconsin’, *Journal of Wildlife Management*, vol. 78, no. 8, pp. 1499–1504, 2014, doi: 10.1002/jwmg.785.

[2] M. O. Hill, ‘Diversity and evenness : A Unifying notation and its consequences’, *Ecology*, vol. 54, no. 2, pp. 427–432, 1973.

[3] H. Petrén, T. G. Köllner, and R. R. Junker, ‘Quantifying chemodiversity considering biochemical and structural properties of compounds with the R package chemodiv’, *New Phytologist*, vol. 237, no. 6, pp. 2478–2492, 2023, doi: 10.1111/nph.18685.

[4] M. Pastore, A. D. L. Pierfrancesco, M. Mingione, and A. Calcagni, ‘Package “overlapping”: Estimation of overlapping in empirical distributions. R package version 2.1.’, 2022.

**SI Tables and Figures**

SI Table 1. Number of scats collected for our study during Summer and Winter for 2020, 2022, and 2023.

| Year | Summer | Winter | Total |
| --- | --- | --- | --- |
| 2020 | 154 | 20 | 174 |
| 2022 | 81 |  | 81 |
| 2023 | 143 | 44 | 187 |
| Totals | 378 | 64 | 442 |

SI Table 2. SIMMR posterior estimates of each diet category for Summer and Winter showing the Mean and standard deviation (SD) for each season and diet category. For each season, SIMMR was run using individual as a random effect to account for multiple whisker segments belonging to a single individual.

|  | Summer | | Winter | |
| --- | --- | --- | --- | --- |
|  | Mean | SD | Mean | SD |
| Small Prey | 0.16 | 0.02 | 0.20 | 0.02 |
| Browsers | 0.32 | 0.04 | 0.62 | 0.04 |
| Human Foods | 0.28 | 0.02 | 0.15 | 0.05 |
| Berries | 0.24 | 0.05 | 0.03 | 0.01 |

SI Table 3. Total number of segments per individual fox during summer and winter collected between 2022 and 2024.

| Fox ID | Summer | Winter |
| --- | --- | --- |
| BF.F.23 | 7 |  |
| F.3.23 | 6 |  |
| F.576 | 5 |  |
| F.881 | 5 |  |
| F2.23 | 4 |  |
| FSC.1 | 5 |  |
| FSC.3 | 7 |  |
| SCF.22 | 8 |  |
| F.1.24 |  | 6 |
| F.226 |  | 7 |
| F.310 |  | 2 |
| F.311.23 |  | 4 |
| F.577 |  | 4 |
| FSC.2 |  | 4 |
| FTBF.22 |  | 7 |
| FXW.23 |  | 7 |
| Totals | 47 | 41 |

SI Table 4. Causes of dietary dissimilarity of foxes (Vulpes vulpes) across seasons.

| Diet item | Average | SD | Ratio | Cumulative Sum |
| --- | --- | --- | --- | --- |
| Berries | 0.20 | 0.01 | 22.79 | 0.28 |
| Insects | 0.16 | 0.01 | 17.01 | 0.51 |
| Moose | 0.09 | 0.01 | 6.08 | 0.64 |
| Birds | 0.08 | 0.01 | 9.37 | 0.75 |
| Hare | 0.07 | 0.02 | 4.15 | 0.85 |
| Squirrel | 0.06 | 0.02 | 3.91 | 0.93 |
| Muskrat | 0.02 | 0.01 | 3.12 | 0.96 |
| Mouse | 0.02 | 0.01 | 1.50 | 0.99 |
| Beaver | 0.01 | 0.00 | 3.27 | 1.00 |

**
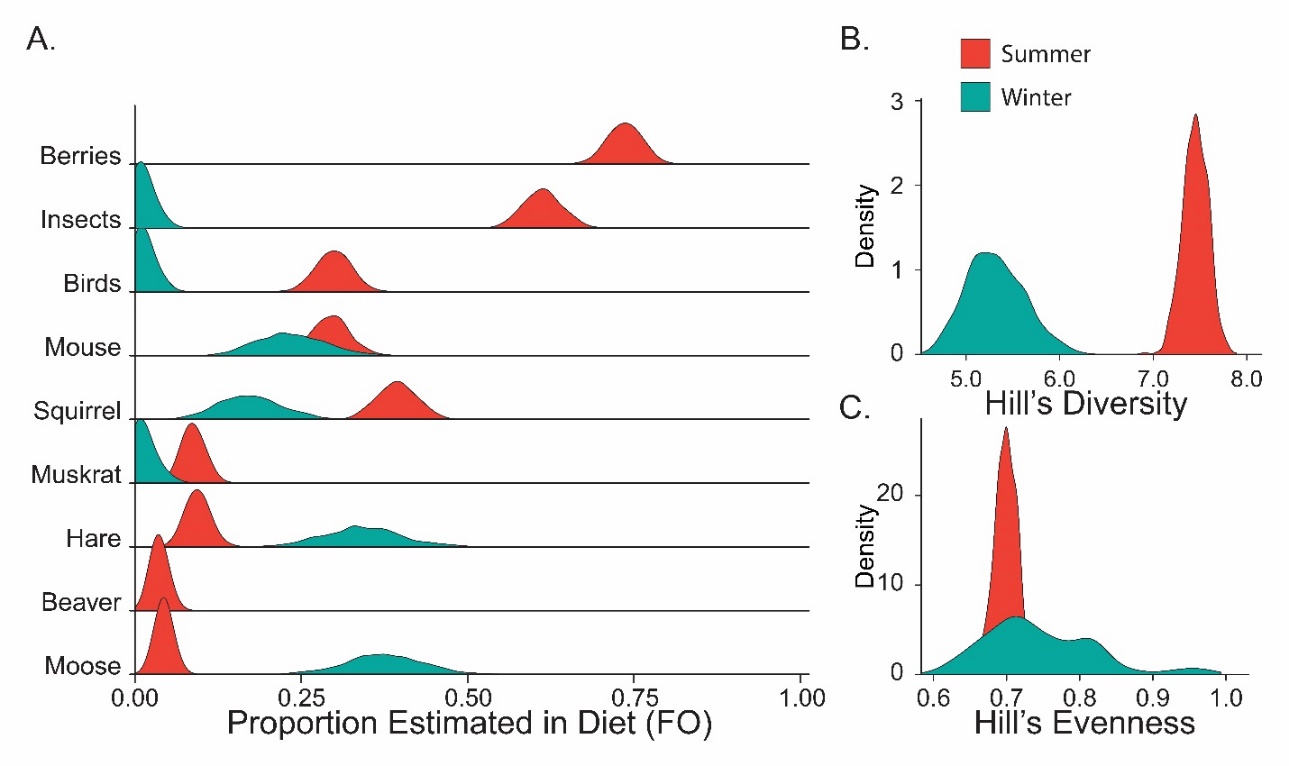
SI Figures**

SI Figure 1. Frequency of occurrence of different items in diet of red fox (*Vulpes vulpes*) during summer (red) and winter (green; A). Hill’s diversity (B) and evenness (C) for summer and winter. Hill’s diversity accounts for the incorporation of diet items, while evenness accounts for the availability of the total resources.


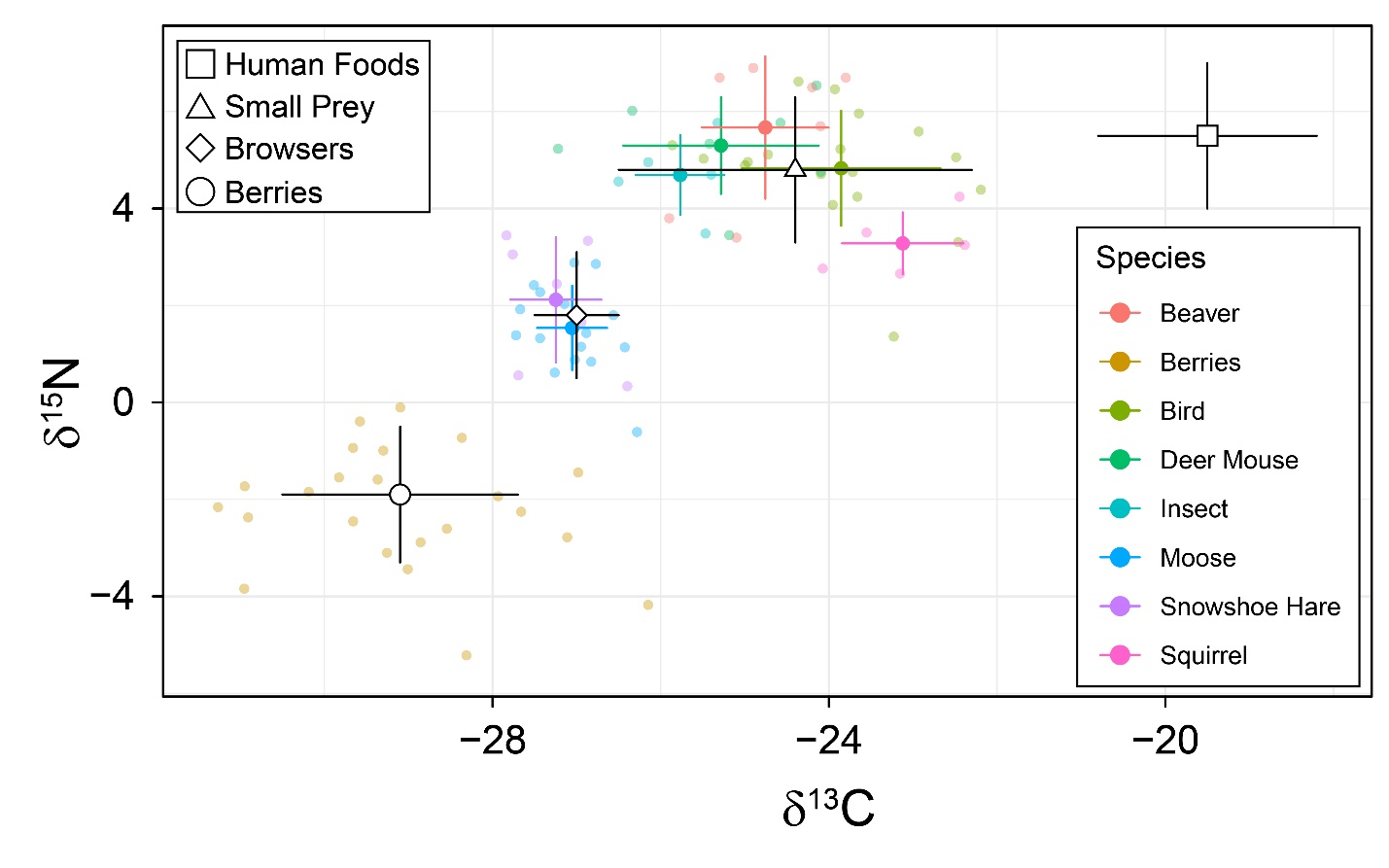


SI Figure 2. Carbon (δ^13^C) and nitrogen (δ^15^N) stable isotope composition of all species (colors) and groups (shapes) included in our analysis. Mean and SD for each species is shown in the corresponding color; note that berries is not visible because the “group” obscures it and is the same.


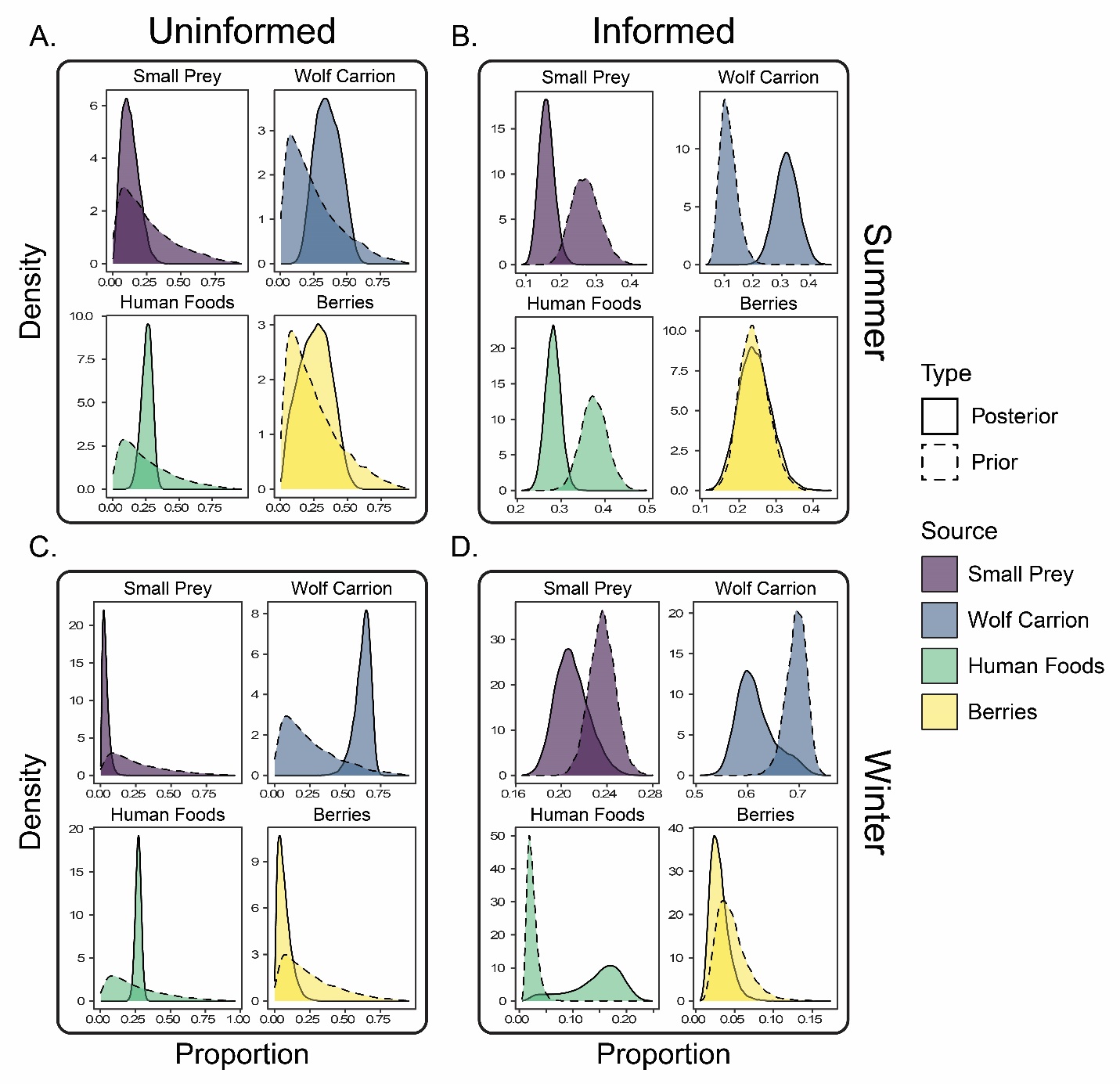
SI Figure 3. Summer (A and B) and Winter (C and D), uninformed (A and C) vs informed (B and D) stable isotope mixing model results for the proportions of small prey (purple), wolf carrion (blue), human foods (green), and berries (yellow). The posterior values are solid lines and the priors are dashed.


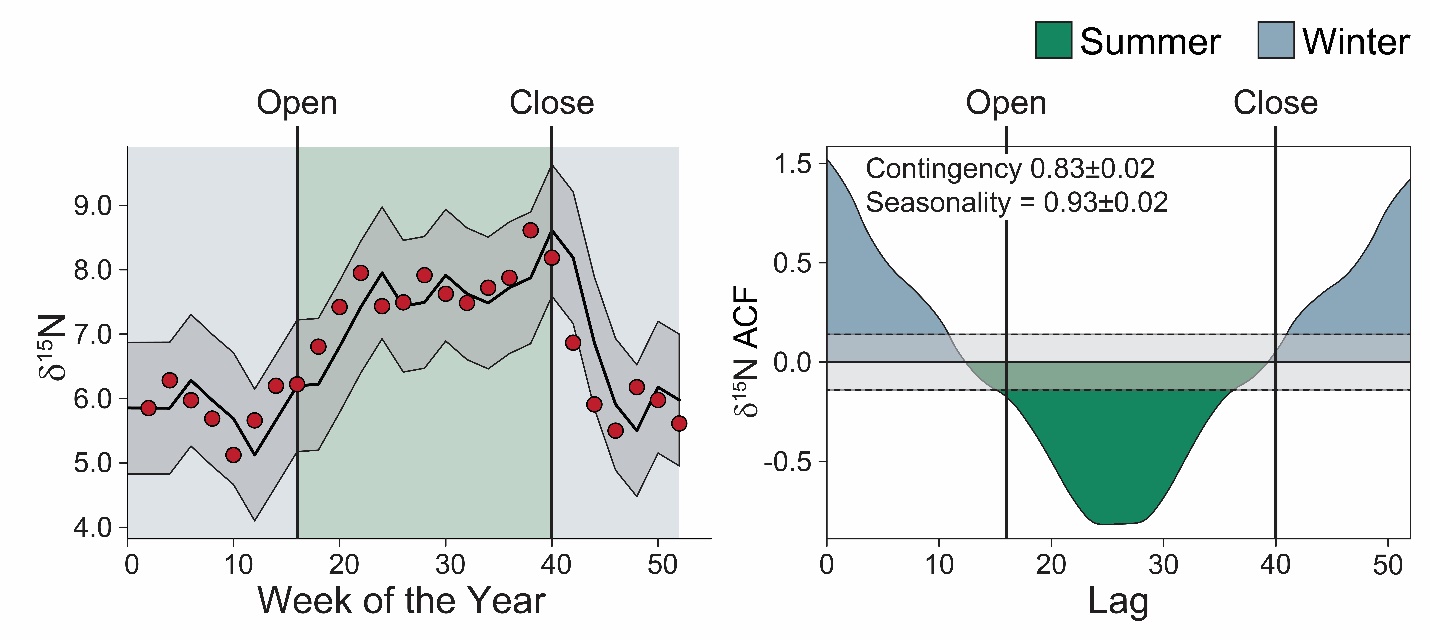
SI Figure 4. (A) Nitrogen stable isotope (δ15N) data of red foxes (*Vulpes vulpes*) collected from whiskers represented as the average value across individuals at the two-week period (red points); the black line with associated 95% confidence intervals are fitted values from our autoregressive model. (B) Seasonal auto-correlation function (ACF) of the yearly reconstructed δ^15^N data of red foxes showing associated positive and negative autocorrelation across winter (blue) and between summer and winter (green), respectively. The dates Isle Royale Opens and Closes are represented as black vertical lines in both figures.
